# The Yield of Essential Oils in *Melaleuca alternifolia* (Myrtaceae) Is Regulated through Transcript Abundance of Genes in the MEP Pathway

**DOI:** 10.1371/journal.pone.0060631

**Published:** 2013-03-27

**Authors:** Hamish Webb, Robert Lanfear, John Hamill, William J. Foley, Carsten Külheim

**Affiliations:** 1 Research School of Biology, Australian National University, Canberra, ACT, Australia; 2 School of Biological Sciences, Monash University, Melbourne, Victoria, Australia; RIKEN Biomass Engineering Program, Japan

## Abstract

Medicinal tea tree (*Melaleuca alternifolia*) leaves contain large amounts of an essential oil, dominated by monoterpenes. Several enzymes of the chloroplastic methylerythritol phosphate (MEP) pathway are hypothesised to act as bottlenecks to the production of monoterpenes. We investigated, whether transcript abundance of genes encoding for enzymes of the MEP pathway were correlated with foliar terpenes in *M. alternifolia* using a population of 48 individuals that ranged in their oil concentration from 39 -122 mg.g DM^−1^. Our study shows that most genes in the MEP pathway are co-regulated and that the expression of multiple genes within the MEP pathway is correlated with oil yield. Using multiple regression analysis, variation in expression of MEP pathway genes explained 87% of variation in foliar monoterpene concentrations. The data also suggest that sesquiterpenes in *M. alternifolia* are synthesised, at least in part, from isopentenyl pyrophosphate originating from the plastid via the MEP pathway.

## Introduction

Plant essential oils are an important crop in many parts of the world and their profitability is closely linked to both the profile and concentration of the oil in each plant. Many of the species grown for essential oils occur as different chemotypes (discontinuous variations in the oil profile: [1]), but selection of the desirable chemotype can be readily monitored by gas chromatography and is rarely a major factor detracting from profitability. In contrast, improving the oil yield of essential oil crops relies on a long process of traditional breeding and in the case of tree crops, this can require many years before production. Recent advances in genomics offer the possibility of identifying the genes and gene variants that are responsible for high yields of essential oils, so significantly shortening the breeding process.

Medicinal tea tree (*Melaleuca alternifolia* Cheel) is a small Myrtaceous tree with sub-dermal foliar oil glands [2] containing a valuable essential oil dominated by monoterpenes [3]. Tea tree oil has wide-ranging antifungal and antibacterial actions and is incorporated into many cosmetic products [4], [5]. Six essential oil chemotypes have been identified in medicinal tea tree [3], [6], but the only one sought by the tea tree industry is that dominated by the monoterpene terpinen-4-ol, which is derived from the spontaneous rearrangement of sabinene hydrate, which in turn is produced by a single terpene synthase [3]. Although the terpinen-4-ol chemotype shows a four-fold variation in oil yield [7] other chemotypes have a higher overall oil concentration. In *M. alternifolia*, the narrow-sense heritability of foliar oil concentration is high (∼0.7) suggesting that genetic control of oil yield is significant [8].

The production of terpenes in plants involves several distinct metabolic pathways and the genes in these pathways have been well studied in model species. The two main pathways, the Mevalonic acid pathway (MVA) in the cytosol and the 2-C-methyl-D-erythritol 4-phosphate pathway (MEP) in the plastid are spatially separated within the cell. A recent review [9]suggests that both pathways generally operate independently. While independence is the “rule” cross-talk and transport of MEP derived isopentyl pyrophosphate (IPP) to the cytosol has been demonstrated on a number of occasions [10], [11], [12], [13], [14]. Monoterpenes, the major constituents of *M. alternifolia* leaf essential oil are synthesised via IPP derived from the MEP pathway, which is likely to have the largest effect on essential oil yield. Potential bottlenecks to flux through the pathway have been identified. In particular, the early steps of the MEPpathway have been identified as constraints to yield of terpene-rich essential oils. Over-expression of 1-deoxy-D-xylulose 5-phosphate reductoisomerase (DXR) in peppermint (*Mentha piperita*) led to plants accumulating 40% more oils in their glandular trichomes relative to the wild-type [15]. Over-expression of DXR and 1-deoxy-D-xylulose 5-phosphate synthase (DXS) in transgenic *Arabidopsis thaliana* expressing Taxadiene synthase (35S:TXS) led to a several fold increased accumulation of taxadiene (a diterpene) over plants just expressing Taxadiene synthase, [16] and over-expression of DXS in tomato, resulted in a 60% increase in isoprenoids [17]. In grape (*Vitis vinifera*), *dxs* co-localizes with a major QTL for the accumulation of three monoterpenes (linalool, nerol and geraniol) [18]. In glandular trichomes of basil (*Ocimum basilicum*) transcript and protein abundance as well as enzyme activity of DXS and DXR correlate with oil yield [19]. While DXS and DXR are the most likely bottlenecks in the MEP pathway, the over-expression of 1-hydroxy-2-methyl-2-(E)-butenyl 4-diphosphate reductase (HDR) in *Arabidopsis* results in a large increase in carotenoids [20]. Other genes may also be important; in *Arabidopsis*, all MEP pathway transcripts are up-regulated upon exposure to light [21] and in Norway spruce (*Picea abies*), four genes that were investigated, *dxs, dxr,* 4-hydroxy-3-methylbut-2-en-1-yl diphosphate synthase (*hds*) and *hdr* were all up-regulated upon induction by a range of treatments [22]. Furthermore, in *Eucalyptus globulus* we discovered several allelic variants in *hds* and *hdr* that associated with foliar concentrations of the monoterpene 1,8-cineole [23]. Based on these results, it is likely that the control of flux through the terpene biosynthesis pathway is controlled at many different levels. Previous work in model plants has provided some clues as to how this may be controlled between individuals in controlled environments, but to date there has been no work into how this variation is controlled in wild populations.

This study investigated the control of quantitative variation in the yield of essential oils in a wild plant population. We have quantified transcript abundance from genes leading to the synthesis of both mono- and sesquiterpenes in leaves from 48 individuals of *M. alternifolia* that vary widely in their concentration of oils.

## Materials and Methods

### Plant Material

**Figure 1:**
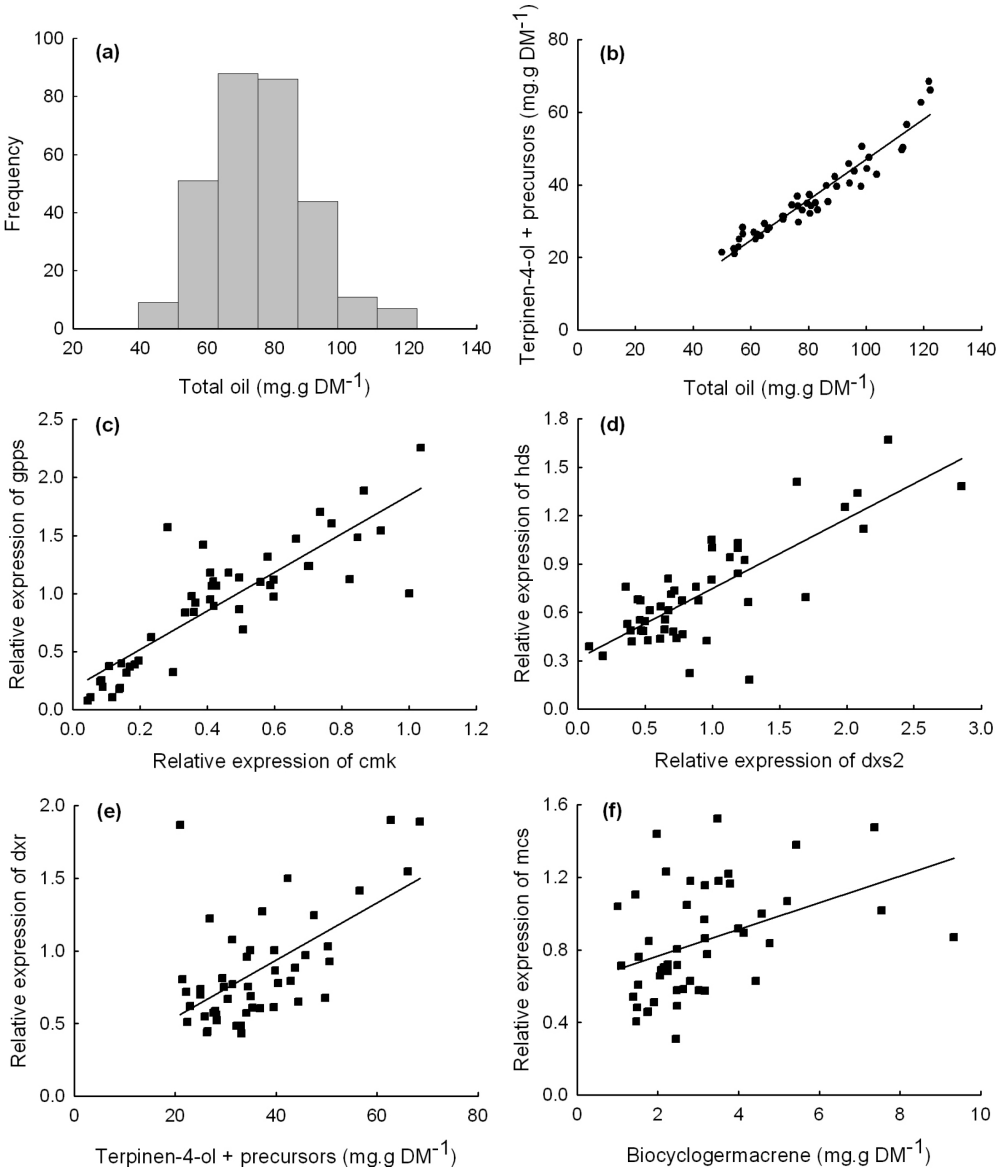
Oil distribution and scatter plots of relationships between essential oil and gene expression parameters. (a) Histogram of total foliar oil concentration in 200 families of M. alternifolia. (b) Scatter plot between foliar concentration of total oil and “terpinen-4-ol +”. Scatter plot showing the correlation between the relative gene expression of (c) cmk and gpps and (d) dxs2 and hds. Scatter plot showing the correlation between the relative gene expression and terpene concentration of (e) dxr and terpinen-4-ol + and (f) mcs and bicyclogermacrene.

Samples from *Melaleuca alternifolia* plants for this study were collected from a New South Wales Department of Primary Industry (NSW DPI) experimental site at Ballina in Northern NSW (28.52.00 S; 153.34.00 E). The site contains plantings of more than 200 families from seed collected from 14 populations within the Clarence River catchment and one population from Port Macquarie. All source populations contain predominantly chemotype 1 individuals in which the terpene profile is dominated by terpinen-4-ol [6], [7]. The foliar oil content of these 200 families is normally distributed (Figure 1a) and we selected 48 individuals (chemotype 1) from 48 families that represented the range of oil yield found within families planted at the site. For each individual, samples of fully expanded foliage of ∼1 year of age were removed for later extraction of terpenes and one branchlet representing the ontogeny of the leaves (from new expanding leaf to mature leaf) was collected and immediately frozen in liquid nitrogen and stored at -80°C for RNA extraction. A “branchlet” covering leaf ontogeny was chosen over mature leaf because terpenes in mature *M. alternifolia* leaf are stored in oil glands which are filled over the ontogeny of the leaf. A branchlet was chosen to “capture” the time when the oils are accumulating and not just maintenance of the mature oil profile.

### Terpene extraction and analysis

Terpenes were extracted with ethanol containing an internal standard of tridecane according to the method described by Russell and Southwell [24] Gas chromatography was carried out on an Agilent 6890 GC using an Alltech AT-35 (35% phenyl, 65% dimethylpolyoxylane) column (Alltech, Wilmington, DE). The column was 60 m long with an internal diameter of 0.25 mm with a stationary phase film thickness of 0.25 μm. Helium was used as a carrier gas. The ethanol extract was filtered through a 0.45 μm filter, and 1 μl was injected at 250°C at a 1:25 split ratio. The temperature program was as follows: 100°C for 5 min, ramping to 200°C at 20°C·min^−1^ followed by a ramp to 250°C at 5°C min^−1^, and held at 250°C for 4 min. The total elution time was 25 minutes. Forty seven components of the solvent extract were identified using an FID and an Agilent 5973 Mass Spectrometer dual setup through an SGE MS/FID splitter. Peaks were identified by comparisons of mass spectra to reference spectra in the National Institute of Standards and Technology library (Agilent Technologies, Deerfield, IL) [25] and major peaks were verified by reference to authentic standards.

### RNA extraction and cDNA synthesis

Leaves were ground to a fine powder in liquid nitrogen. Total RNA was extracted from leaves with Ambion RNAqueous kit (Applied Biosystems, Foster City, CA) with the addition of sodium isoascorbate (Sigma-Aldrich, Sydney, Australia) to saturation. After the first wash step, DNase (Promega, Madison, WI) was added to the wash column and incubated for 30 min at room temperature. RNA quantity and quality was first determined by separating 5 μl of the RNA extract on a 1% Agarose gel containing ethidium bromide in 1x TAE buffer (Figure S1) and measurements of 1 μl on a NanoDrop ND-1000 spectrophotometer (Thermo Scientific, Wilmington, DE).Absorption ratios of A260/A280 were between 1.8 and 2.0. First strand cDNA synthesis was performed using the Moloney Murine Leukemia Virus Reverse Transcriptase (Promega, Madison, WI), which was anchored with a T_30_VN primer according to the manufacturer. All samples were diluted to 25 ng.µl^−1^ with MilliQ H_2_O and used as template for real time PCR.

### Primer design and Quantitative Real time PCR

Homologues of *Arabidopsis thaliana* genes of MEP and mevalonate (MVA) pathways were obtained and their amino acid sequences used in a tblastn search against the Genbank (http://www.ncbi.nlm.nih.gov/) *Melaleuca alternifolia* EST database. For genes which did not have a homologue in the *M. alternifolia* EST database, primers which were designed for *Eucalyptus globulus* [26] were used to amplify homologues by PCR reaction. For the remainder, primers were designed for homologues from the *Eucalyptus grandis* genome sequence (http://www.phytozome.net/eucalyptus.php), then amplified and together with all other amplicons sequenced on an AB3700 sequencer using standard protocols. All *M. alternifolia* sequences were aligned to *Arabidopsis thaliana, Eucalyptus grandis* and *E. globulus* genomic sequences. Gene and species-specific primers for the reference genes {elongation factor 1α (*ef1α*), tubulin alpha1 (*tua*)}, MEP pathway genes {(1-deoxy-D-xylulose 5-phosphate reductase (*dxr*), 1-deoxy-D-xylulose 5-phosphate synthase (*dxs1, dxs2, dxs3*), 4-diphosphocytidyl-2-C-methyl-D-erythritol synthase (*mct*), 4-diphosphocytidyl-2-C-methyl-D-erythritol kinase (*cmk*), 2-C-methyl-D-erythritol 2,4-cyclodiphospate synthase (*mcs*), (E)-4-Hydroxy-3-methyl-but-2-enyl pyrophosphate synthase (*hds*)}, mevalonate pathway {(3-hydroxy-3-methylglutaryl-CoA synthase (*hmgs1*), mevalonate kinase (*mvk*), 5-pyrophospho-mevalonate decarboxylase (*pmd1*)} and downstream terpenoid pathway {isopentyl pyrophosphate isomerase (*ippi1, ippi2*) and geranyl pyrophosphate synthase (*gpps*)} were designed such that one or both primers crossed an intron or alternatively, that the desired amplicon would cross one or more introns. Products from each of the primer sets were sequenced to verify the identity of the amplified gene. Genes from gene families- e.g. *dxs,* were named in accordance to their sequence similarity with *Arabidopsis thaliana* sequences e.g. *Arabidopsis dxs1, dxs2* and *dxs3* in Arabidopsis are most similar to *dxs1*, *dxs2* and *dxs3*, respectively, in *M. alternifolia*. Primer efficiencies for each primer pair were determined through a dilution series and all values were between 1.95 and 1.99 with the exception of gpps, which had a value of 1.88. *Ef1* and *tua* were chosen as reference genes after picking a number of commonly used house keeping genes that had also been identified as good reference genes in *E. globulus* [27], [28], [29], [30] . We tested the expression stability of these genes across our samples and chose *ef1* and *tua* as the best reference genes for this study. All primers used for the generation of these data are listed in Table S1. Gene transcript abundance was quantified using the Fluidigm Biomark platform (Fluidigm, South San Francisco, CA) with EvaGreen™ (Bio-Rad, Gladesville, NSW, Australia) according to the Fluidigm protocol, using 15 cycles of pre-amplification. The quantification of the transcript *dxs1* failed consistently in the Fluidigm experiment and was thus excluded from all analysis. Three technical replicates were performed for each gene and individual. The C_T_ values and standard deviation of each sample are all shown in Table S2. Given the design of the study, which used a common garden trial, it was not possible to use biological replicates, given each tree represented a unique genotype grown in the same environment. Transcript abundance was calculated using the Fluidigm Real-Time PCR analysis software (Fluidigm, South San Francisco, CA) using both *ef1α* and *tua* as internal standards. The ratio between both internal standards was stable between individuals and their average was used.

### Correlation and multiple regression analysis

Pairwise correlation analysis was performed between each terpene trait as well as between transcript abundance traits and terpene traits using GenStat 12^th^ edn (VSN International, Hemel Hempstead, UK). Multiple regression analysis was used to test the relationship between transcript abundance and oil traits using the statistical package R [31]. This analysis used the most abundant monoterpene terpinen-4-ol (including its monoterpene precursors sabinene, *cis-* and *trans-*sabinene hydrate) and the most abundant sesquiterpene, bicyclogermacrene, as response variables.

Multiple regression analysis followed Crawley [32]; (i) The distribution of each variable was analyzed, and rows of data where an individual had one or more values which appeared to be erroneous were excluded; (ii) A linear model which included all predictor variables (i.e. abundance data from all transcripts) was used to start the analysis; (iii) Non-linearity in the relationships between predictor and response variables were checked for by fitting models that included squared terms of each variable; (iv) Pairwise interactions between predictors were checked by fitting models that included all possible pairwise interactions between variables in the model. Since the number of predictors is large relative to the number of data points, there is a risk of over-fitting the model. Because of this, fitting any models in which there were fewer than three data-points per predictor were avoided [32]. For steps (iii) and (iv), this involved fitting a series of models that included small numbers of squared or interaction terms in randomly chosen groups.

Significant squared and interaction terms from steps (iii) and (iv) were included in the full model along with all untransformed predictor variables. Stepwise model selection using the Akaike Information Criterion was then used to simplify the full model, using the MASS package in R [33]. This final model was then checked using standard linear model diagnostics in R. In particular, data points with Cook's distance >0.5 were considered as potential outliers, and analyses were repeated without these data points. Results of analyses both including and excluding potential outliers are presented. All analysis scripts written in R are available from the authors.

## Results

### Quantitative and qualitative analysis of essential oils

Gas chromatography – mass spectroscopy revealed the presence of 20 monoterpenes and 27 sesquiterpenes, of which 18 and 11, respectively, could be identified by comparison to reference mass spectral data [25] and authentic standards. Total terpene yield ranged from 39.4 -122.3 mg.g DM^−1^, (mean 75.5 mg.g DM^−1^) (Figure 1a). Because terpinen-4-ol is derived from spontaneous re-arrangements of *cis-* and *trans-*sabinene hydrate as well as sabinene, we summed the concentrations of these separate monoterpenes to form a trait we called “terpinen-4-ol plus precursors” (hereafter “terpinen-4-ol +”). This component dominated the total oil profile and ranged from 14.5 – 68.5 mg.g DM^−1^ (mean 32.2 mg.g DM^−1^). The most abundant sesquiterpene was bicyclogermacrene which ranged from 1.0 – 9.3 mg.g DM^−1^ (mean 3.1 mg.g DM^−1^). Linear regression showed that there was a high degree of correlation between individual components of the oil with the highest correlation being between, “terpinen-4-ol +” and total oil (*R^2^* = 0.922) (Figure 1b). There was also high correlation between (i) the most abundant sesquiterpene (bicyclogermacrene) and the sum of all sesquiterpenes (*R^2^* = 0.678), (ii) within monoterpenes (α-pinene and terpinolene; *R^2^* = 0.593) and (iii) within sesquiterpenes (δ-cadinene and bicyclogermacrene; *R^2^* = 0.664) (Table S3).

### Transcript abundance from genes in the terpenoid biosynthesis pathway

The relative abundance of transcripts for *dxr, dxs2*, *dxs3*, *cmk, mcs, mct* and *hds* from the MEP pathway, *mvk, hmgs1* and *pmd1* from the MVA pathway, *ippi1* and *ippi2* as well as *gpps* (which acts downstream of *ippi* in the pathway that leads to monoterpene formation) was quantified. The comparison of transcript abundance between genes showed that there were high levels of correlation within each pathway, with lower degrees of correlation between pathways, with the exception of *mct*, which was not correlated with any other genes (Table S4). The highest correlation observed was between *gpps* and *cmk* (*R^2^* = 0.725) (Figure 1c). Within the MEP pathway, there were strong correlations between *hds* and *dxs2* (Figure 1d), and *hds* and *cmk* (*R^2^* = 0.611 and 0.61, respectively). Within the MVA pathway the highest correlation was between *pmd* and *mvk* (*R^2^* = 0.526), while between the MVA and MEP pathways the highest correlation was between *mvk* and *dxr* (*R^2^* = 0.322).

### Correlation of gene expression with quantitative variation of terpene traits

The degree of correlation between the relative transcript abundance of *dxr*, *dxs2*, *dxs3, mcs, cmk, mct, hds*, *ippi1*, *ippi2*, *mvk*, *hmgs1, pmd1* and *gpps* was tested against several different terpene traits using simple regression (Table S5). Many MEP pathway genes such as *dxs, dxr, cmk, mcs* and *hds* were correlated with total oil yield and “terpinen-4-ol +” concentrations. A scatter plot of the relation between the foliar concentration of “terpinen-4-ol +” and the relative expression of *dxr* (*R^2^* = 0.337) is shown in Figure 1e. The highest degree of correlation between foliar sesquiterpenes and gene expression was between bicyclogermacrene and *mcs* (*R^2^* = 0.152) (Figure 1f). Notably, the relative expression of *dxr, mcs* and *cmk* was also correlated with the concentration of bicyclogermacrene - a sesquiterpene. The expression of *ippi2* was correlated with the concentration of total foliar sesquiterpenes as well as the ratio of mono- to sesquiterpenes, suggesting it may have a role in resource allocation.

**Figure 2:**
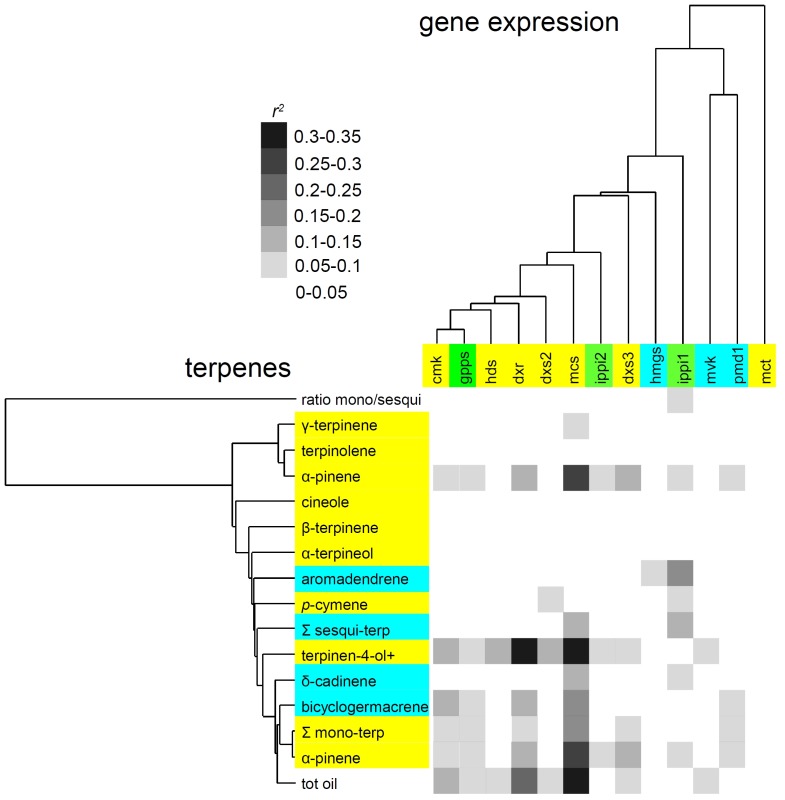
Cluster analysis of terpene traits and gene expression and correlation matrix between terpene traits and gene expression. Genes of the MEP pathway are shown in yellow, those of the MVA pathway in blue. Downstream genes ippi and gpps are shown in green. Monoterpenes are shown in yellow and sesquiterpenes are shown in blue.

A cluster analysis was used to describe the relationships amongst terpenes and the expression of all genes in the MEP pathway (Figure 2). Genes from the MEP pathway (with the exception of *mct*) cluster together with *gpps* and *ippi2*, whereas there were no clusters amongst the foliar terpenes. A correlation matrix between transcript abundance and quantitative terpene data is shown (Figure 2).

We used a multiple regression model to test whether the genes expressed in the chloroplastic MEP pathway (*dxs2, dxs3, dxr, mct, cmk, mcs, hds* plus *gpps*) were significant predictors of the foliar concentration of *“*terpinen-4-ol +” (Table 1). Examination of the data suggested that one measurement from *dxs3* was erroneous. This datapoint (id CMA15) was removed before any further analysis. The final model contained six linear terms, two squared terms and six pairwise interaction terms. No further outliers were identified.

**Table 1:** Final model for prediction of terpinen-4-ol concentrations from transcript levels of MEP pathway genes.

Predictor	Slope	*P*-value
dxs2	−70.883	0.001
dxr	37.682	0.018
mct	−4.129	<0.001
mcs	53.857	0.18
hds	−9.499	0.585
gpps	−8.3	0.019
I(hdˆ2)	−92.068	<0.001
I(mcˆ2)	−41.043	0.125
mct:gpps	3.603	<0.001
dxr:mcs	−77.482	0.018
dxr:hds	54.508	0.01
hds:I(mcˆ2)	60.705	0.014
dxs2:I(mcˆ2)	−52.135	0.017
dsx2:mcs	145.007	0.001
*R^2^*	0.87	
Model *P*-value	<0.001	

A second multiple regression model was constructed to test whether genes from the MEP pathway in the chloroplast (*dxs2, dxs3, dxr, mct, cmk, mcs, hds,* plus *gpps*) and genes from the MVA pathway in the cytosol (*hmds, mvk*, and *pmd*) were significant predictors of bicyclogermacrene concentrations (Table 2). The initial model had one outlier, and so the model was re-derived (including testing for non-linearity in variables, and testing for significant interaction terms) after removing this value. The terms included in the final model (nine linear terms and three interaction terms), did not differ when this outlier was excluded (Table 2).

**Table 2:** Two models (with and without outlier) for prediction of bicyclogermacrene concentrations from transcript levels of MEP and MVA pathway genes.

	all datapoints		outlier removed	
Predictor	Slope	*P*-value	Slope	*P*-value
dxs2	0.050	0.970	−1.708	0.206
dxs3	−1.159	0.219	−1.198	0.159
dxr	3.457	0.099	6.472	0.004
cmk	−6.413	0.048	−12.013	0.001
mcs	6.570	0.011	7.398	0.002
hds	5.312	0.169	12.472	0.005
pmd1	2.391	0.131	2.503	0.080
mvk	−2.685	0.081	−2.205	0.111
hmgs	1.809	0.093	1.228	0.208
mcs:hds	−6.619	0.084	−9.575	0.009
dsx2:cmk	7.077	0.019	12.352	<0.001
dsx2:hds	−3.448	0.067	−6.972	0.001
*R^2^*		0.507		0.603
Model *P*-value		0.007		0.001

In order to investigate the relative contributions of MEP and MVA genes to foliar bicyclogermacrene concentrations, two submodels of the final model between gene expression and foliar bicyclogermacrene concentration were constructed. The first of the two submodels included only those predictors from the final model which are expressed in the chloroplast (i.e. MEP pathway genes *dxs2, dxs3, dxr, cmk, mcs, hds,* and the interaction terms *mcd:hds, dxs2:cmk, dxs2:hds*). The second submodel included only those predictors in the final model which are expressed in the cytosol (MVA pathway genes *pmd1, mvk, hmgs*). In other words, we tested whether genes from both the cytosol and the chloroplast contributed to variation in foliar bicyclogermacrene. Both of these models were compared to the full model using a likelihood ratio test implemented in the *lmtest* package in R [34]. The likelihood ratio test suggested that both submodels fitted the observed variation in bicyclogermacrene concentrations significantly worse than the full model (Table 3). This suggests that MVA pathway genes in the cytosol and MEP pathway genes in the chloroplast both make significant contributions to the final synthesis of bicyclogermacrene. However, the results also show that the expression data from the genes located in the chloroplast explain far more of the variance in bicyclogermacrene concentrations (*R^2^* = 0.500) than do the genes from the cytosol (*R^2^* = 0.146) (Table 3).

**Table 3:** Likelihood ratio test between two submodels and the full model that predict bicyclogermacrene concentrations.

Model	lnL	*R^2^*	*P* (LTR)
bicyclogermacrene final model	−67.358	0.603	n/a
bicyclogermacrene chloroplast component	−72.845	0.500	0.012
bicyclogermacrene cytosol component	−84.970	0.146	<0.001

## Discussion

Quantitative variation in the yield of terpene-dominated essential oils is widespread and of significant ecological and economic importance. Terpene yield is under strong genetic control in woody plants [35], [36]. Although the genes involved in the two biosynthetic pathways leading to the formation of terpenes (MEP and MVA pathways) are well-known in crops and *Arabidopsis*, understanding the genetic basis of quantitative variation of terpene traits in woody plants provides opportunities for rapid improvements in yield and better returns to growers given the long breeding cycles required for trees. In this study we investigated the genetic control of terpene yield from the Australian Myrtaceae species *Melaleuca alternifolia*.

The first step of the MEP pathway, which catalyses the conversion of pyruvate and D-glyceraldehyde 3-phosphate to 1-deoxyxylylose 5-phosphate (DXS), has been the focus of most studies to date [21] and was the first step of the pathway that was discovered [37]. Much evidence has accumulated to show that this enzyme is the rate-limiting step of the pathway (reviewed in Introduction). Some reports have focused on the gene expression of all genes in the MEP pathway upon abiotic stimulus, finding for example that light induces all seven steps of the pathway and that each gene is under the control of a circadian signal [21] or that sucrose induced gene expression in the pathway [38]. We focused on the expression of these genes in a natural population of plants with a large sink (foliar terpenes that accumulate in the glands) for the MEP pathway. Our results indicate that a general signal exists that regulates the expression of each pathway gene with the exception of *mct*. Either *mct* is not regulated through this signal or our assay was non-functional.

The first aim of this study was to identify correlations between the transcript abundance of candidate genes and terpene traits. Our analysis revealed strong co-expression of genes within the MEP pathway of the chloroplast. Five out of seven transcripts from this pathway (plus geranyl pyrophosphate synthase, *gpps*) had similar relative transcript abundances. The expression of one gene, *mct*, did not correlate with any other genes and *dxs3*, one of the three copies of 1-deoxy-D-xylulose 5-phosphate synthase did not cluster with other MEP pathway genes (Figure 2). This suggests *dxs3*, which is likely in a different clade, is not directly involved in foliar monoterpene biosynthesis and is performing a different function within the plant. The pattern of expression suggests that there is a common factor that regulates transcription within the MEP pathway and extends the list of genes that have a significant impact on yield of terpenes (*dxs, dxr, hdr, gpps*) [15], [16], [20], [22]. Within the cytosolic MVA pathway, co-expression was less striking, but still significant and in addition, we observed correlations in the expression of genes between the MEP and MVA pathways albeit to a lesser extent than within each pathway (Table S3).

Several studies (reviewed in the Introduction) have shown that the expression of *dxr* and *dxs* have a strong influence on terpenoid oil yield. Our results show pairwise correlations between individual genes within the MEP pathway and foliar “terpinen-4-ol +” for all genes bar *mct* (Figure 2). Given the correlation of the genes within the MEP pathway, we cannot say which genes may or may not be more important in controlling flux through the pathway. It also means that individual gene to trait correlations are of limited predictive utility. This raises the question as to whether *dxs* and *dxr* are such significant “bottlenecks” in the MEP pathway as has previously been supposed. Although it is clear from other studies that both *dxs* and *dxr* are significantly correlated with terpene yield, ours is the first analysis of the whole pathway and it is possible that the more extensive patterns of co-expression might be found with a similar broader analysis of other systems. It is possible that *dxs* and *dxr* could act either as bottlenecks or their over-expression could initiate regulatory cascades that result in the up-regulation of other transcripts within the pathway. Drawing strong conclusions about the mechanisms that control flux in *M. alternifolia* from our data are further complicated by the fact *dxs* is post transcriptionally regulated in other species [15] and that protein levels don't always relate to transcript abundance. While the data makes it clear that transcript levels of the genes within the MEP pathway have an effect on yield in *M. alternifolia*, the exact mechanisms that lead to this result are not obvious at this stage.

The multiple regression approach employed here gives a much more realistic picture than single gene regressions of the importance of interactions between multiple genes in both the MEP and MVA pathways and the variations in the yield of mono- and sesquiterpenes. The results show that transcript abundance of MEP pathway transcripts explains a large amount of the variation in foliar oil yield we observed. Given that the oil profile of *M. alternifolia* is dominated by monoterpenes, the variations in MEP pathway genes are the most important determinant of overall oil yield and in particular the yield of components (“terpinen-4-ol +”) that are sought by industry. This indicates that expression differences between individuals are likely to be an important determinant in differing oil yields.

Our results also provide strong evidence that sesquiterpenes in *M. alternifolia* are, at least in part, synthesized from IPP derived from the MEP pathway. Unidirectional transport of IPP from the plastid to the cytosol has been demonstrated using labeled precursors in a number of other species such as spinach, kale and Indian mustard [39], snapdragon [10] and *Arabidopsis* [40], but not in tomato trichomes [41]. The amount of IPP that is transported differs widely in those species that have been examined. For example in snapdragon, IPP from the plastid is used for the biosynthesis of 100% of sesquiterpenes, while in *Catharanthus roseus* only small amounts of plastid derived IPP are used in sesquiterpene synthesis [42]. In order to show what proportion of IPP from the chloroplast is incorporated into sesquiterpenes, experiments with labeled intermediates for both the MVA and MEP pathway need to be undertaken [10]. Our data does suggest that a significant amount of IPP originates from the chloroplast with explanatory values for correlation of MVA genes being about three times lower than for MEP pathway genes and there was no evidence of any IPP transport from the cytosol to the chloroplast.

These results pave the way for further improving essential oil yield in medicinal tea tree. Because of the low linkage disequilibrium in forest trees, a candidate gene approach in association mapping to detect alleles in genes of the MEP and MVA pathway will be an important first step. However, the extent of co-regulation of MEP pathway genes means that selection made on only one or two alleles might be insufficient to drive sustained increases in oil yield. Identifying the putative transcriptional regulators that control the whole pathway will be necessary to understand how to select for increased flux through the whole MEP pathway and how this translates into greater yield for the benefit of the tea tree industry.

The Australian Myrtaceae *Melaleuca alternifolia* is an ideal study object for the control of terpene yield. Our collection of 200 individuals showed a three-fold variation in total terpene yield and more than four-fold variation of the major constituent terpinen-4-ol. In order to identify the regulatory mechanisms of this variation, we quantified transcripts abundance in 48 individuals from the MEP and MVA pathways as well as genes that act downstream towards the biosynthesis of terpenes. Our data show that the expression of genes from the MEP pathway, as well as *gpps* is positively correlated to monoterpene yield and that the expression of these genes is strongly inter-correlated. Both MEP and MVA pathway genes are predictors of sesquiterpene concentration, providing evidence for export of isopentyl diphosphate from the chloroplast to the cytosol in this species.

## Supporting Information

Figure S1 Agarose gel images showing separation of RNA from 48 samples of *M. alternifolia* used in this study.
(TIF)

Table S1 List of diagnostic primers for quantitative real-time PCR.
(XLS)

Table S2 C_T_ values for each sample and diagnostic primer pair with standard deviation.
(XLS)

Table S3 Simple linear regression analysis between terpene traits. Adjusted R^2^ and corresponding probabilities are shown. ^*^  =  P<0.05, ^**^  =  P<0.01, ^***^  = P<0.001.
(XLS)

Table S4 Simple linear regression analysis between transcript abundance of genes from the MEP and MVA pathways. Adjusted R^2^ and corresponding probabilities are shown. ^*^  = P<0.05, ^**^  = P<0.01, ^***^  = P<0.001.
(XLS)

Table S5 Simple linear regression analysis between terpene traits and transcript abundance. Adjusted R^2^ and corresponding probabilities are shown. ^*^  = P<0.05, ^**^  = P<0.01, ^***^  = P<0.001.
(XLS)
