# Supplementary figures and images for: The Yield of Essential Oils in Melaleuca alternifolia (Myrtaceae) Is Regulated through Transcript Abundance of Genes in the MEP Pathway
Source: PLoS One. 2013 Mar 27;8(3):e60631. doi: 10.1371/journal.pone.0060631 (PMC3609730; doi:10.1371/journal.pone.0060631)

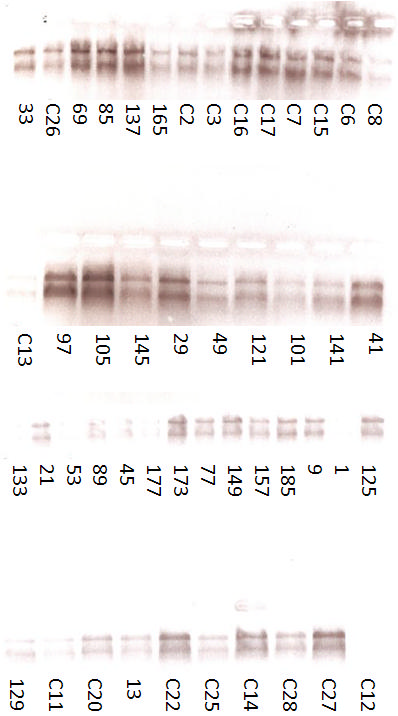

Supplement: Figure S1 — Agarose gel images showing separation of RNA from 48 samples of M. alternifolia used in this study. (TIF) [file pone.0060631.s001.tif]
